# Analytical solutions for cavity contraction in strain-softening materials with linear or exponential strength decay

**DOI:** 10.1038/s41598-024-65186-y

**Published:** 2024-07-01

**Authors:** Maria Anthi, Thomas Pferdekämper, Georgios Anagnostou

**Affiliations:** ETH Zurich, Zurich, Switzerland

**Keywords:** Ground response curve, Tunnelling, Strain-softening, Strength decay, Tresca, Mohr–Coulomb, Engineering, Civil engineering

## Abstract

This paper presents an analytical investigation into the contraction of spherical and cylindrical cavities excavated in strain-softening rock masses obeying the Mohr–Coulomb or Tresca yield criterion, with linear or exponential uniaxial compressive strength decay. The derivation of the ground response curves is based on the simplifying assumption that the strains inside the plastic zone are completely plastic. This significantly simplifies the mathematical formulation, enabling the derivation of closed-form solutions. An alternative simplifying approach which partially neglects the elastic strains inside the plastic zone and which is commonly adopted in the literature, is also examined. The accuracy of the simplified solutions is evaluated by comparing their predictions with rigorous solutions obtained by numerical finite-difference analyses. The investigation demonstrates that the proposed closed-form solutions represent a significant improvement on those based on the commonly-made simplifying assumption involving partial neglect of elastic strains.

## Introduction

The contraction of cavities excavated in strain-softening rock masses has so far been studied using analytical, semi-analytical and numerical methods, which can generally be categorized into two groups based on their treatment of strain within the plastic zone: rigorous methods utilizing incremental plasticity and simplified methods. Recent works and comprehensive reviews of the state of the art can be found in^1–11^.

From a theoretical perspective, the rigorous methods are robust, but expressing closed-form solutions for the cavity contraction problem has proven challenging. Instead, numerical integration is typically required. In practice, for arbitrary yield conditions and flow rules, the system of the governing ordinary differential equations can only be solved numerically, using for example the Runge–Kutta algorithm^12–14^. In general, these methods are too complex for practical use, due to the need to solve intricate second-order (or systems of) differential equations or to implement advanced, tailor-made constitutive models in existing finite element codes. Closed-form solutions are by far easier to apply in engineering design computations, but exist only for specific constitutive models and boundary value problems as can be seen from Table 1, which provides an overview of existing analytical methods of determining the relationship between cavity wall displacement and support pressure (“Ground response curve”, GRC) for perfectly plastic and softening materials. Closed-form solution exist only for problems exhibiting rotational symmetry, that is circular tunnel-cross sections under plane strain conditions and spherical cavities.

**Table 1:** Overview of analytical solutions.

Yield condition	Treatment of the elastic strains in the plastic zone	Perfectly plastic material		Softening material with linear decay law		Softening material with exponential decay law	
		Circular hole in plane strain	Spherical hole	Circular hole in plane strain	Spherical hole	Circular hole in plane strain	Spherical hole
Mohr–Coulomb	Rigorous	✓^(c)^	✓^(c)^	–	–	–	–
	Simplified A^(a)^	✓^(d)^	–	✓^**(e), (i)**^	**–**^**(i)**^	**–**^**(i)**^	**–**^**(i)**^
	Simplified B^(b)^	✓^(f)^	✓^(h)^	**–**^**(i)**^	**–**^**(i)**^	**–**^**(i)**^	**–**^**(i)**^
Tresca	Rigorous	✓^(c)^	✓^(c)^	✓^(g)^	✓^(g)^	–	–
	Simplified A^(a)^	–	–	**–**^**(i)**^	**– **^**(i)**^	**– **^**(i)**^	**– **^**(i)**^
	Simplified B^(b)^	✓^(h)^	✓^(h)^	**– **^**(i)**^	**– **^**(i)**^	**– **^**(i)**^	**– **^**(i)**^

^(a)^Neglecting the elastic strains that develop during plastic yielding.

^(b)^Neglecting completely the elastic strains in the plastic zone.

^(c)^Salençon^15^.

^(d)^Panet^16^.

^(e)^Panet^16^, for the special case of zero residual cohesion.

^(f)^Kovári^17^.

^(g)^Carranza-Torres^18^.

^(h)^Yu and Rowe^19^.

^(i)^This paper.

Regarding the constitutive model, rigorous closed-form solutions exist for perfect plasticity, but only for a special case of softening, the Tresca material with linear strength decay^18^. This paper aims to fill this gap by providing a series of easy-to-use closed-form solutions for geomaterials that undergo strength reduction during shearing. The cases analysed are highlighted in bold in Table 1. Both cylindrical tunnels (represented by the circular hole model at plane strain) and spherical cavities are considered. Plane strain models, while simplifying reality by not accounting for the accurate stress-path followed by the ground during the advance of the tunnel face (see^20^), are widely employed in tunnelling practice. This is because they allow for an intuitive and straightforward analysis of the ground-support interaction, known as the convergence-confinement method^21,22^, and provide insights into the behaviour of tunnel and surrounding rock far behind the face. Spherical cavities are of importance as an approximation of the conditions in front of the tunnel heading^23^, providing insights into the development of ground deformation around the advancing tunnel face. In addition to the Tresca flow criterion (which is valuable as a model for clays under undrained conditions), the Mohr–Coulomb criterion is considered, which is more suitable for geomaterials under dry or drained conditions. The analysis of two strength decay laws (linear or exponential) allows a better representation of the observed softening behaviour.

**Figure 1:**
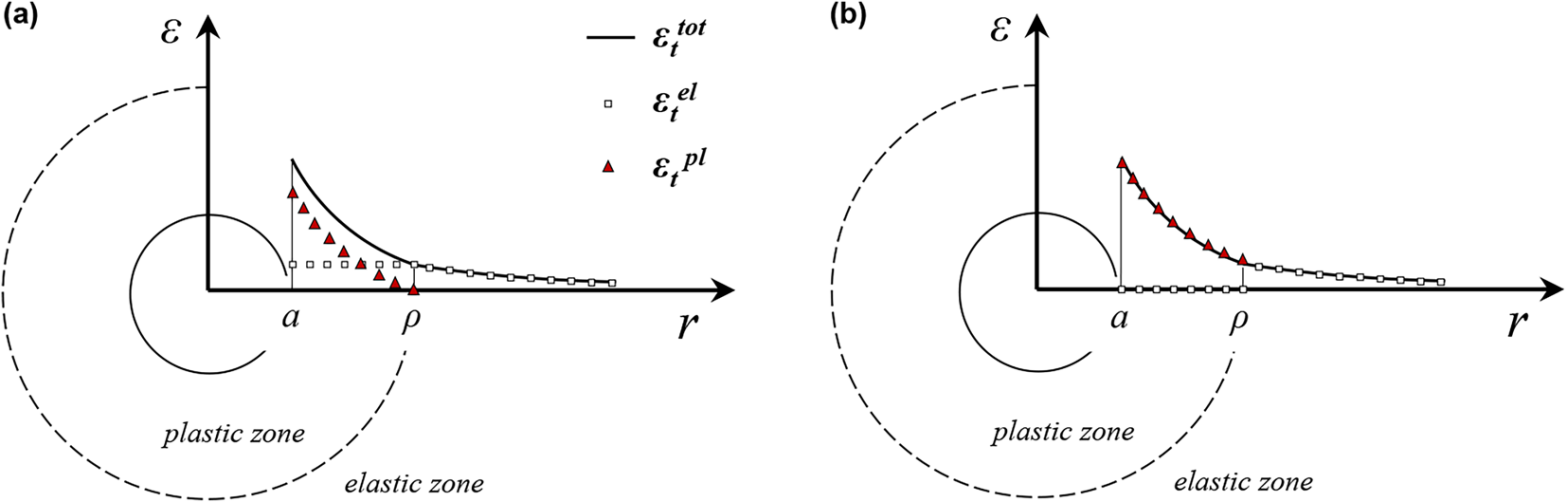
Radial distribution of tangential strain, elastic tangential strain and plastic tangential strain, (**a**), neglecting the elastic strains that develop during plastic yielding and, (**b**), neglecting completely the elastic strains inside the plastic zone.

The mathematical treatment can be significantly simplified by neglecting the elastic strains that develop during plastic yielding, *i.e*. assuming that the total strain inside the plastic zone is equal to the sum of the elastic strain at the elasto-plastic boundary and the plastic strain (Fig. 1a). Adopting this simplifying assumption (hereafter referred to as “Assumption A”) reduces the mathematical complexity considerably (see^24^), thus enabling closed form solutions. Panet^16^ employed this simplification to derive closed-form Ground Response Curves (GRCs), for the specific case of a Mohr–Coulomb (MC) strain-softening material with zero residual strength. However, Guan et al.^25^ conducted a comparative study on MC materials with constant friction angle and linear decay of cohesion and dilation angle in the strain softening regime, demonstrating that this simplification underestimates the radial cavity wall displacement by 20–40% compared to rigorous numerical methods (see^14^). Furthermore, it can be proven that this simplification is incompatible with the zero volumetric strain constraint for materials following the Tresca yield criterion, in the case of spherical cavities, resulting in even more significant displacement underestimations (see Section “Error of the simplified solutions” of this paper).

Kovári^17^ and Yu and Rowe^19^ derived GRCs for perfectly-plastic MC materials by completely neglecting the elastic strains within the plastic zone (Fig. 1b). This simplification (hereafter referred to as “Assumption B”) does not violate the incompressibility condition for Tresca materials, whether in relation to cylindrical or spherical cavities. However, it fails to ensure a continuous distribution of plastic strains and, assuming that strength in the strain-softening regime is controlled by the plastic strain, of strength, too. Specifically, disregarding completely the elastic strains in the plastic zone in combination with the condition of displacement continuity at the elastoplastic interface *r* = *ρ* results in a discontinuity in the plastic strain field; the plastic tangential strain at *r* = *ρ*^+^ is by definition equal to zero while at *r* = *ρ*^–^ it is equal to the elastic tangential strain at *r* = *ρ*^+^, that is *ε*_*t,ρ*_^*el*^. This discontinuity already results in a sudden drop in strength at *r* = *ρ*.

In this paper, starting with the definition of the problem (Section “Problem statement”), we adopt the aforementioned simplifying assumptions A and B (Fig. 1a and b, respectively) to derive in Section “Derivation” closed-form GRCs both for cylindrical and spherical cavities excavated in elasto-plastic strain-softening materials obeying the MC or Tresca yield criterion with exponential or linear strength decay (cases in bold in Table 1). Section “Synopsis of the ground response equations” summarizes and presents uniformly the derived equations. Subsequently, an interesting aspect of the predicted behaviour (the interplay between deformational behaviour and stability) and the effect of the softening law on the ground response are discussed (Section “Discussion”). Finally, Section “Error of the simplified solutions” shows that the solutions derived adopting Kovári’s simplification^17^ (Fig. 1b) are, in most cases and particularly for spherical cavities, considerably more accurate than those derived under the simplification of Fig. 1a.

## Problem statement

**Figure 2:**
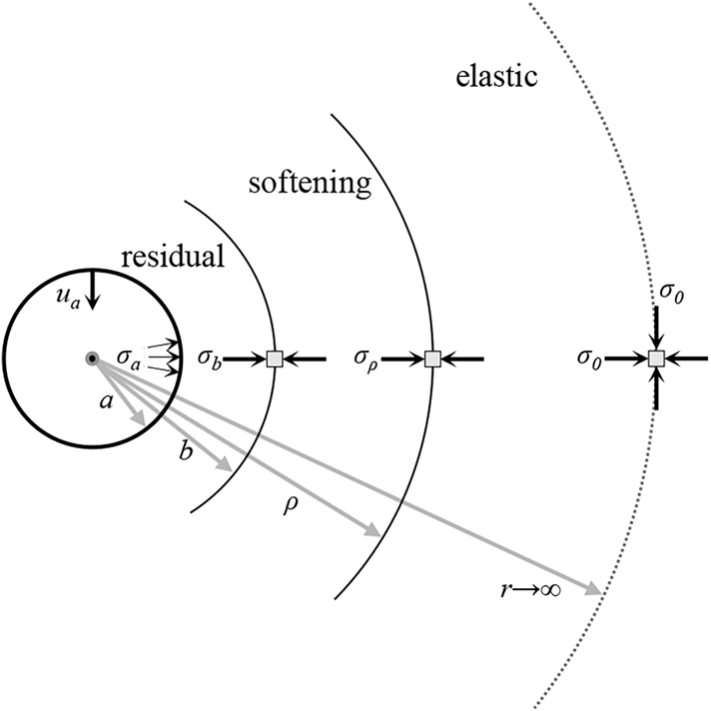
Problem setup and zones of the stress field in a strain softening rock mass.

We consider a cylindrical or spherical cavity with radius *a* located in an infinite, homogeneous, and isotropic medium (Fig. 2). As in most GRC computations, the in-situ stress field is assumed to be isotropic and homogeneous. Solutions for anisotropic *in-situ* stress fields have been derived only for perfectly plastic materials^31–33^. The assumption of isotropic *in-situ* stress is reasonable for deep tunnels^34^ and sufficiently accurate for principal stress differences up to 30%^35^.

The cavity is supported by a uniform pressure *σ*_*a*_, while the far field stresses are equal to the *in-situ* stress *σ*_*0*_. Under these assumptions the system fulfils rotational symmetry. We are looking for the relationship between the support pressure *σ*_*a*_ and the radial displacement *u*_*a*_ of the cavity wall.

**Figure 3:**
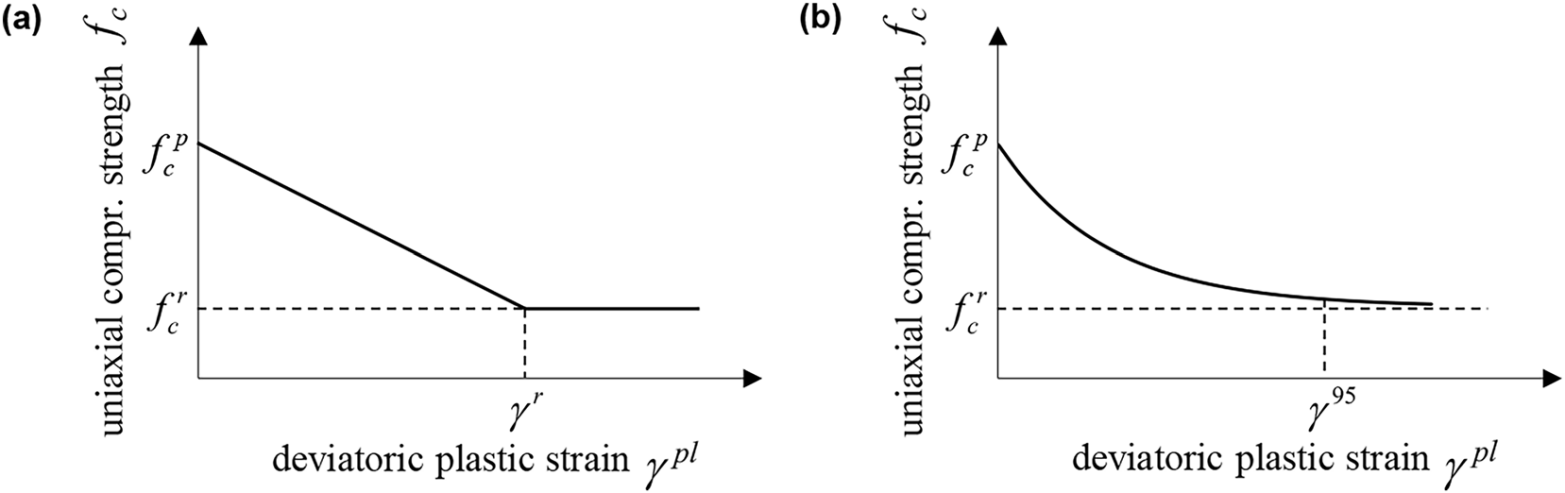
Strength over deviatoric plastic strain for, (**a**), linear and, (**b**), exponential strength decay.

The rock mass is taken as a linearly elastic, softening plastic material obeying the Hooke’s law and the MC or Tresca yield criterion with linear or exponential strength decay (Fig. 3a and b, respectively) and constant friction and dilation angles. For the sake of simplicity, the possibility of out-of-plane plastic flow is not considered (see^27,31^ and^32^ for investigations into the effect of out-of-plane plastic flow in perfectly plastic materials and^1,2^ for the case of softening materials).

Following^14^ and^26^ we assume that the decrease in strength is controlled by the deviatoric plastic strain *γ*^*pl*^, which is defined as follows:

1 $$\gamma^{pl} = \varepsilon_{t}^{pl} - \varepsilon_{r}^{pl} ,$$

where $\varepsilon_{t}^{pl}$ and $\varepsilon_{r}^{pl}$ represent the tangential and radial plastic strains, respectively.

In the case of linear strength decay (Fig. 3a):

2 $$f_{c} = \left\{ {\begin{array}{*{20}l} {f_{c}^{p} - \frac{{\gamma^{pl} }}{{\gamma^{r} }}(f_{c}^{p} - f_{c}^{r} ) = f_{c}^{p} \left( {1 - \delta \frac{{\gamma^{pl} }}{{\gamma^{r} }}} \right),} \hfill & {{\text{if}}\;\gamma^{pl} < \gamma^{r} ,} \hfill \\ {f_{c}^{r} = f_{c}^{p} (1 - \delta ),} \hfill & {{\text{if}}\;\gamma^{pl} > \gamma^{r} ,} \hfill \\ \end{array} } \right.$$

where *f*_*c*_, $f_{c}^{p}$ and $f_{c}^{r}$ denote the uniaxial compressive strength and its peak and residual values, respectively; *δ* is the maximum decrease in strength, normalized by the peak strength; and *γ*^*r*^ is the deviatoric plastic strain at which the residual strength is attained. For exponential decay (Fig. 3b), Einav and Randolph’s function^28^ is adopted:

3 $$f_{c} \left( {\gamma^{pl} } \right) = f_{c}^{p} - \left( {f_{c}^{p} - f_{c}^{r} } \right)\left( {1 - e^{{{{ - 3\gamma^{pl} } \mathord{\left/ {\vphantom {{ - 3\gamma^{pl} } {\gamma^{95} }}} \right. \kern-0pt} {\gamma^{95} }}}} } \right) = f_{c}^{p} \left( {1 - \delta \left( {1 - e^{{{{ - 3\gamma^{pl} } \mathord{\left/ {\vphantom {{ - 3\gamma^{pl} } {\gamma^{95} }}} \right. \kern-0pt} {\gamma^{95} }}}} } \right)} \right),$$

where *γ*^*95*^ is the deviatoric plastic strain at which the strength reduction equals 95% of the difference between peak and residual value*.*

## Derivation

Upon cavity unloading, the rock mass remains initially elastic; the deviatoric stresses increase with decreasing support pressure and, at a certain support pressure *σ*_*ρ,*_ may become so high that the stress path at the cavity wall reaches the yield surface and rock starts experiencing irreversible deformations.

The elasticity solutions for a spherical or cylindrical cavity in combination with the condition that the stress state (*σ*_*r*_*, σ*_*t*_) at the cavity wall fulfils the yield condition with the peak strength parameters lead to the following expressions for the radial stress and for the strains at the tunnel wall at the onset of plastification (cf., e.g.,^29^):

4 $$\sigma_{\rho } = \frac{{\left( {1 + \zeta } \right)\sigma_{0} - \zeta f_{c}^{p} }}{\zeta m + 1},$$

5 $$\varepsilon_{t,\rho }^{el} = - \frac{{\varepsilon_{r,\rho }^{el} }}{\zeta } = \frac{{\sigma_{0} \left( {m - 1} \right) + f_{c}^{p} }}{E}\frac{1 + \nu }{{\zeta m + 1}},$$

where *m* is a function of the friction angle (= 1 and (1 + sin*φ*)/(1-sin*φ*) for the Tresca and MC material, respectively); *E* and *ν* denote the Young’s modulus and the Poisson’s ratio, respectively; and *ζ* is the symmetry parameter (= 1 for cylindrical cavities and 2 for spherical cavities).

Further cavity unloading results to the formation of a zone of radius *ρ* where the rock mass experiences plastic shearing, and its strength decreases according to the softening laws of Fig. 3. In the case of linear strength decrease, the deviatoric plastic strain at the excavation boundary may reach *γ*^*r*^ at a certain support pressure *σ*_*b*_; further cavity unloading (*σ*_*a*_ < *σ*_*b*_) will then result in the formation of an inner plastic zone up to a radius *b*, where rock strength equals residual strength.

So, in the case of linear decay the system consists in general of three zones, hereafter referred to as the elastic zone (an infinite body with a cylindrical or spherical cavity), the softening and residual zone (thick-walled cylinders or spheres), and, in the case of exponential decay, the system consists of an outer elastic zone and an inner softening zone (Fig. 2).

The GRC can be determined by considering the aforementioned system components separately, taking account of the radial stress and radial displacement continuity conditions at their interfaces.

As mentioned in the Introduction, the simplifying Assumption A neglects the elastic strains that develop during plastic yielding, but takes account of the elastic strains that develop up to the onset of yielding (that is $\varepsilon _{t}^{{pl}} \, = \,\varepsilon _{t} - \varepsilon _{{t,\rho }}^{{el}} ,\varepsilon _{r}^{{pl}} \, = \,\varepsilon _{r} - \varepsilon _{{r,\rho }}^{{el}}$), whereas the simplifying Assumption B considers the entire strain inside the plastic zone as being plastic, that is it completely disregards the elastic strains (*ε*_*t*_^*pl*^ = *ε*_*t*_, *ε*_*r*_^*pl*^ = *ε*_*r*_). The derivation of the GRC will be illustrated considering the case of the MC yield criterion with linear strength decay and Assumption B. The solution for Assumption A can be derived analogously.

### Kinematic considerations

Neglecting the elastic strains in the plastic zone allows the deformations to be analysed solely from the flow rule (*ε*_*r*_^*pl* ^+ *ζ κ ε*_*t*_^*pl*^ = 0, where *κ* = (1 + sin*ψ*)/(1 − sin*ψ*) and *ψ* denotes the dilatancy angle) and the kinematic relationships, that is without making use of equilibrium and yield conditions^16,17,19^.

The assumption about the elastic strains in combination with the kinematic relationships and the plastic flow rule results in a differential equation for the radial displacement, the solution of which provides the displacement field in the plastic zone in the form *u*(*r/ρ*). So, in the case of Assumption B considered in this derivation,

6 $$\frac{{u_{a} }}{a} = \left( {\frac{\rho }{a}} \right)^{\zeta \,\kappa + 1} \varepsilon_{t,\rho }^{el} .$$

After we have determined the displacement field, we obtain from the kinematic relationships the radial and tangential strains and from Eq. (1) the plastic shear strain to be introduced in the softening law:

7 $$\gamma^{pl} = \left( {1 + \zeta \,\kappa \;} \right)\left( {\frac{\rho }{r}} \right)^{\zeta \,\kappa + 1} \varepsilon_{t,\rho }^{el} .$$

Note that the form of the equations for the radial displacement of the wall and the distribution of the plastic deformation (Eqs. 6 and 7) depend on which simplifying assumption is made (Eqs. 6 and 7 hold for Assumption B), but not on the yield condition or the softening law. The latter are important for the relationship between radius *ρ* and support pressure, which is determined below based upon static considerations.

### Static considerations

Introducing the yield condition

8 $$\sigma_{t} = m \cdot \sigma_{r} + f_{c} \left( {\gamma^{{_{pl} }} } \right),$$

where *σ*_*r*_ and *σ*_*t*_ denote the radial and the tangential stress, respectively, into the equilibrium condition

9 $$\frac{{d\sigma_{r} }}{dr} = \zeta \cdot \frac{{\sigma_{t} - \sigma_{r} }}{r}$$

results in a differential equation for the radial stress field *σ*_*r*_(*r*),

10 $$\frac{{d\sigma_{r} }}{dr} - \zeta \cdot \frac{{\sigma_{r} \left( {m - 1} \right) + f_{c} \left( {\gamma^{{_{pl} }} } \right)}}{r} = 0$$

with *γ*^*pl*^ depending on *r*/*ρ* (Eq. 7), the solution of which for the boundary condition *σ*_*r*_(*a*) = *σ*_*a*_ provides the stress field in the form *σ*_*r*_ = *f*(*r/ρ, σ*_*a*_). This equation in combination with the condition *σ*_*r*_(*ρ*) = *σ*_*ρ*_ results in a relationship between *σ*_*a*_ and *ρ*, the specific form of which depends on the kinematic simplification (the aforementioned Assumption A or B), the yield criterion and the softening law.

### Up to formation of the residual zone

Considering the initial (linear decay) part of the linear relation, the integration of Eq. (10) yields the following *σ*_*a*_(*ρ*)—relationship:

11 $$\sigma_{a}^{{}} = \frac{1 + \zeta }{{1 + \zeta m}}\left( {\frac{\rho }{a}} \right)^{{ - \zeta \left( {m - 1} \right)}} \left( {\sigma_{0}^{{}} + \frac{{f_{c}^{p} }}{m - 1}} \right) - \frac{{f_{c}^{p} }}{m - 1} - \frac{{\delta \,f_{c}^{p} \;\varepsilon_{t,\rho }^{el} }}{{\gamma^{r} }}\;\frac{{\zeta \left( {1 + \zeta \kappa } \right)}}{{1 + \zeta \left( {m + \kappa - 1} \right)}}\left( {\left( {\frac{\rho }{a}} \right)^{1 + \zeta \kappa } - \left( {\frac{\rho }{a}} \right)^{{ - \zeta \left( {m - 1} \right)}} } \right).$$

### Onset of the residual zone

Equation (11) holds provided that there is no residual zone yet, which means that the plastic shear strain at the tunnel wall is less then *γ*^*r*^. This is the case (see Eq. 7) as long as the normalized radius of the plastic zone is less than a critical value *ω*:

12 $$\frac{\rho }{a}\; \le \;\omega = \max \left\{ {1,\;\,\left( {\frac{{\gamma^{r} }}{1 + \zeta \,\kappa }\frac{1}{{\varepsilon_{t,\rho }^{el} }}} \right)^{{\frac{1}{1 + \zeta \,\kappa }}} } \right\}.$$

The support pressure at the onset of the formation of the residual zone (that is *σ*_*b*_) can be obtained from Eq. (11) with *ρ/a* = *ω*.

The introduction of the constraint “*ω* ≥ 1” in Eq. (12) is associated with the aforementioned discontinuity in the plastic strain field: If *γ*^*r*^ is smaller than (1 + *ζ κ*) $\varepsilon_{t,\rho }^{el}$, then the plastic strain that is «instantaneously» imposed at *r* = *ρ* (Fig. 1b) suffices to cause formation of a residual zone right from the start, i.e.* b* = *ρ*. Τhat is for *ρ/a* > 1, and consequently *ω* (which according to the last r.h.s. term of Eq. 12 would become less than 1) must be enforced to the value 1.

### After formation of the residual zone

If *σ*_*b*_ > 0, then for *σ*_*a*_ < *σ*_*b*_ (or *ρ/a* > *ω*) a residual zone develops. The outer radius of the residual zone (*b*) is such that the support pressure exerted by the residual zone upon the softening zone equals to *σ*_*b*_ (and consequently *ρ/b* = *ω*). It can be obtained from the bearing capacity equation of a fully plastified thickwalled cylinder or sphere that exhibits a uniform strength (the residual strength *f*_*c*_^*r*^), is loaded at its outer boundary by *σ*_*b*_ and is supported at its inner boundary by *σ*_*a*_ (see, *e.g*.,^17^):

13 $$\frac{b}{a} = \left( {\frac{{\sigma_{b} + \frac{{f_{c}^{r} }}{m - 1}}}{{\sigma_{a} + \frac{{f_{c}^{r} }}{m - 1}}}} \right)^{{\frac{1}{{\zeta \left( {m - 1} \right)}}}} .$$

The support pressure for *ρ/a* > *ω* is obtained from Eq. (13) and considering that *ρ/b* = *ω*, it reads as follows:

14 $$\sigma_{a} = \;\;\left( {\sigma_{b} + \frac{{f_{c}^{r} }}{m - 1}} \right)\;\;\left( {\frac{\omega }{{{\rho \mathord{\left/ {\vphantom {\rho a}} \right. \kern-0pt} a}}}} \right)^{{\zeta \left( {m - 1} \right)}} - \frac{{f_{c}^{r} }}{m - 1}.$$

### Closure

The GRC can be computed analytically by considering the displacement *u*_*a*_ as an independent parameter, first computing *ρ* from Eq. (6) and finally *σ*_*a*_ from Eqs. (11) or (14).

The derivation for the other cases (yield condition, softening law and assumptions about strains inside the plastic zone) follows the same path, the only difference being in the integration results. The next section summarizes the final equations in nondimensional form for all four combinations of yield function and softening law and the simplifying assumptions A and B.

## Synopsis of the ground response equations

The relationship between radius of the plastic zone and cavity wall displacement reads as follows:

15 $$\hat{u}_{a} > \hat{\varepsilon }_{t,\rho }^{el} :\;\;\;\;\tilde{\rho } = \left\{ {\begin{array}{*{20}l} {\left( {\frac{1 + \zeta \,\kappa }{2}\frac{{\hat{u}_{a} }}{{\hat{\varepsilon }_{t,\rho }^{el} }} + \frac{1 - \zeta \,\kappa }{2}} \right)^{{\frac{1}{\zeta \,\kappa + 1}}} } \hfill & {\text{(Assumption A),}} \hfill \\ {\left( {\frac{{\hat{u}_{a} }}{{\hat{\varepsilon }_{t,\rho }^{el} }}} \right)^{{\frac{1}{\zeta \,\kappa + 1}}} } \hfill & {\text{(Assumption B),}} \hfill \\ \end{array} } \right.$$

where

16 $$\hat{u}_{a} = \frac{{u_{a} }}{a}\tilde{E},$$

17 $$\hat{\varepsilon }_{t,\rho }^{el} = \left\{ {\begin{array}{*{20}l} {\left( {m - 1} \right)\frac{1 + \nu }{{1 + \zeta m}}} \hfill & {\text{(MC yield criterion),}} \hfill \\ {\tilde{f}_{c}^{p} \frac{1.5}{{1 + \zeta }}} \hfill & {({\text{Tresca yield criterion),}}} \hfill \\ \end{array} } \right.$$

$\tilde{\rho }$ is the normalized radius of the plastic zone ($\tilde{\rho } = \rho /a \ge 1$); $\tilde{E}$ and $\tilde{f}_{c}^{p}$ are the normalized Young’s modulus and peak uniaxial compressive strength, respectively, defined as follows:

18 $$\tilde{E} = \frac{E}{{\overline{\sigma }_{0} }},\quad \tilde{f}_{c}^{p} = \frac{{f_{c}^{p} }}{{\overline{\sigma }_{0} }},$$

where the denominator is the *in-situ* stress (in the case of Tresca criterion) or its Caquot-transformation (in the case of MC yield criterion):

19 $$\overline{\sigma }_{0} = \left\{ {\begin{array}{*{20}l} {\sigma_{0} + \frac{{f_{c}^{p} }}{m - 1}} \hfill & {\text{(MC yield criterion),}} \hfill \\ {\sigma_{0} } \hfill & {{\text{(Tresca yield criterion)}}{.}} \hfill \\ \end{array} } \right.$$

The expressions for the support pressure depend on the specific combination of yield condition and softening law. They are given below using the normalization:

20 $$\tilde{\sigma }_{...} = \left\{ {\begin{array}{*{20}l} {\frac{{\sigma_{...} + \frac{{f_{c}^{p} }}{m - 1}}}{{\sigma_{0} + \frac{{f_{c}^{p} }}{m - 1}}}} \hfill & {\text{(MC yield criterion),}} \hfill \\ {\frac{{\sigma_{...} }}{{\sigma_{0} }}} \hfill & {{\text{(Tresca yield criterion)}}{.}} \hfill \\ \end{array} } \right.$$

### MC yield criterion with linear strength decay

21 $$1 \le \tilde{\rho } \le \omega :\;\;\;\;\tilde{\sigma }_{a} = \left\{ {\begin{array}{*{20}l} {\frac{1 + \zeta }{{1 + \zeta m}}\;\tilde{\rho }^{\zeta - \zeta m} + \mu \frac{2}{m - 1}\;\left( {\frac{{\left( {1 + \zeta \kappa } \right)\tilde{\rho }^{\zeta - \zeta m} + \zeta \left( {m - 1} \right)\tilde{\rho }^{1 + \zeta \kappa } }}{{1 + \zeta \left( {m + \kappa - 1} \right)}} - 1} \right)} \hfill & {\text{(Assumption A),}} \hfill \\ {\frac{1 + \zeta }{{1 + \zeta m}}\tilde{\rho }^{\zeta - \zeta m} + \mu \;\frac{{\zeta \left( {1 + \zeta \kappa } \right)}}{{1 + \zeta \left( {m + \kappa - 1} \right)}}\left( {\tilde{\rho }^{1 + \zeta \kappa } - \tilde{\rho }^{\zeta - \zeta m} } \right)} \hfill & {\text{(Assumption B),}} \hfill \\ \end{array} } \right.$$

22 $$\tilde{\rho } > \omega :\;\;\;\tilde{\sigma }_{a}^{{}} = \left( {\tilde{\sigma }_{b}^{{}} - \frac{{\delta \tilde{f}_{c}^{p} }}{m - 1}} \right)\;\left( {\frac{{\tilde{\rho }}}{\omega }} \right)^{\zeta - m\zeta } + \frac{{\delta \tilde{f}_{c}^{p} }}{m - 1},$$

with

23 $$\omega = \left\{ {\begin{array}{*{20}l} {\left( {1 + \frac{{\gamma^{r} \tilde{E}}}{{2\hat{\varepsilon }_{t,\rho }^{el} }}} \right)^{{\frac{1}{\zeta \,\kappa + 1}}} } \hfill & {\text{(Assumption A),}} \hfill \\ {\max \left\{ {1,\;\,\left( {\frac{1}{1 + \zeta \,\kappa }\frac{{\gamma^{r} \tilde{E}}}{{\hat{\varepsilon }_{t,\rho }^{el} }}} \right)^{{\frac{1}{1 + \zeta \,\kappa }}} } \right\}} \hfill & {\text{(Assumption B),}} \hfill \\ \end{array} } \right.$$

24 $$\mu = \frac{{\delta \tilde{f}_{c}^{p} }}{{\gamma^{r} \tilde{E}}}\;\;\hat{\varepsilon }_{t,\rho }^{el} ,$$

$\hat{\varepsilon }_{t,\rho }^{el}$ after Eq. (17) and $\tilde{\sigma }_{b}^{{}}$ after Eq. (21) for $\tilde{\rho } = \omega$.

An inspection of these equations readily reveals that the GRC can be expressed as follows:

25 $$\tilde{\sigma }_{a} = f\left( {\hat{u}_{a} ,\;\delta \tilde{f}_{c}^{p} ,\;\frac{{\gamma^{r} \tilde{E}}}{{\delta \tilde{f}_{c}^{p} }}\;,\;\zeta ,\;m,\;\kappa ,\,\;\nu \,} \right).$$

### Tresca yield criterion with linear strength decay

26 $$1 \le \tilde{\rho } \le \omega :\;\;\tilde{\sigma }_{a} = \left\{ {\begin{array}{*{20}l} {1 - \frac{\zeta }{\zeta + 1}\tilde{f}_{c}^{p} - \zeta \tilde{f}_{c}^{p} \,\ln \tilde{\rho } + 2\,\mu \,\zeta \,\,\left( {\frac{{\tilde{\rho }^{1 + \zeta } - 1}}{1 + \zeta } - \ln \tilde{\rho }} \right)} \hfill & {{\text{(Assumption A)}},} \hfill \\ {1 - \frac{\zeta }{\zeta + 1}\tilde{f}_{c}^{p} - \zeta \tilde{f}_{c}^{p} \,\,\ln \tilde{\rho } + \mu \;\zeta \;\left( {\tilde{\rho }^{1 + \zeta } - 1} \right)} \hfill & {\text{(Assumption B),}} \hfill \\ \end{array} } \right.$$

27 $$\tilde{\rho } > \omega :\;\;\tilde{\sigma }_{a} = \tilde{\sigma }_{b} - \zeta \tilde{f}_{c}^{p} \left( {1 - \delta } \right)\ln \frac{{\tilde{\rho }}}{\omega },$$

with *ω* after Eq. (23) and $\tilde{\sigma }_{b}^{{}}$ after Eq. (26) for $\tilde{\rho } = \omega$.

### MC yield criterion, with exponential strength decay

28 $$\tilde{\sigma }_{a} = \left\{ {\begin{array}{*{20}l} {\frac{1 + \zeta }{{1 + \zeta m}}\;\tilde{\rho }^{\zeta - m\zeta } + \delta \tilde{f}_{c}^{p} \left( {\frac{{1 - \tilde{\rho }^{\zeta - m\zeta } }}{m - 1} + \,\zeta \,e^{2\lambda } \frac{{E_{n} \left( {2\lambda \tilde{\rho }^{1 + \zeta \kappa } } \right) - \tilde{\rho }^{\zeta - m\zeta } E_{n} \left( {2\lambda } \right)}}{1 + \zeta \kappa }} \right)} \hfill & {({\text{Assumption A}}),} \hfill \\ {\frac{1 + \zeta }{{1 + \zeta m}}\;\;\tilde{\rho }^{\zeta - m\zeta } + \delta \tilde{f}_{c}^{p} \left( {\frac{{1 - \tilde{\rho }^{\zeta - m\zeta } }}{m - 1} + \,\,\zeta \frac{{E_{n} \left( {\left( {1 + \zeta \kappa } \right)\lambda \tilde{\rho }^{1 + \zeta \kappa } } \right) - \tilde{\rho }^{\zeta - m\zeta } E_{n} \left( {\left( {1 + \zeta \kappa } \right)\lambda } \right)}}{1 + \zeta \kappa }} \right)} \hfill & {({\text{Assumption B}}),} \hfill \\ \end{array} } \right.$$

where

29 $$\lambda = \frac{3}{{\gamma^{95} \tilde{E}}}\hat{\varepsilon }_{t,\rho }^{el} ,$$

30 $$n = 1 + \frac{\zeta - m\zeta }{{1 + \zeta \kappa }}$$

and *E*_*n*_() is the generalized exponential integral function.

### Tresca yield criterion with exponential strength decay

31 $$\tilde{\sigma }_{a} = \left\{ {\begin{array}{*{20}l} {1 - \frac{{\zeta \tilde{f}_{c}^{p} }}{\zeta + 1} - \zeta \tilde{f}_{c}^{p} \,\ln \tilde{\rho } + \delta \tilde{f}_{c}^{p} \,\zeta \left( {\ln \tilde{\rho } + \,e^{2\lambda } \frac{{{\text{Ei}}\left( { - 2\lambda } \right) - {\text{Ei}}\left( { - 2\lambda \tilde{\rho }^{1 + \zeta } } \right)}}{1 + \zeta }} \right)} \hfill & {({\text{Assumption A}}),} \hfill \\ {1 - \frac{{\zeta \tilde{f}_{c}^{p} }}{\zeta + 1} - \zeta \tilde{f}_{c}^{p} \ln \tilde{\rho } + \delta \tilde{f}_{c}^{p} \;\zeta \left( {\ln \tilde{\rho } + \frac{{{\text{Ei}}\left( { - \lambda \left( {1 + \zeta } \right)} \right) - {\text{Ei}}\left( { - \lambda \,\left( {1 + \zeta } \right)\tilde{\rho }^{1 + \zeta } } \right)}}{1 + \zeta }} \right)} \hfill & {({\text{Assumption B}}),} \hfill \\ \end{array} } \right.$$

where *Ei*() is the exponential integral function.

## Discussion

**Figure 4:**
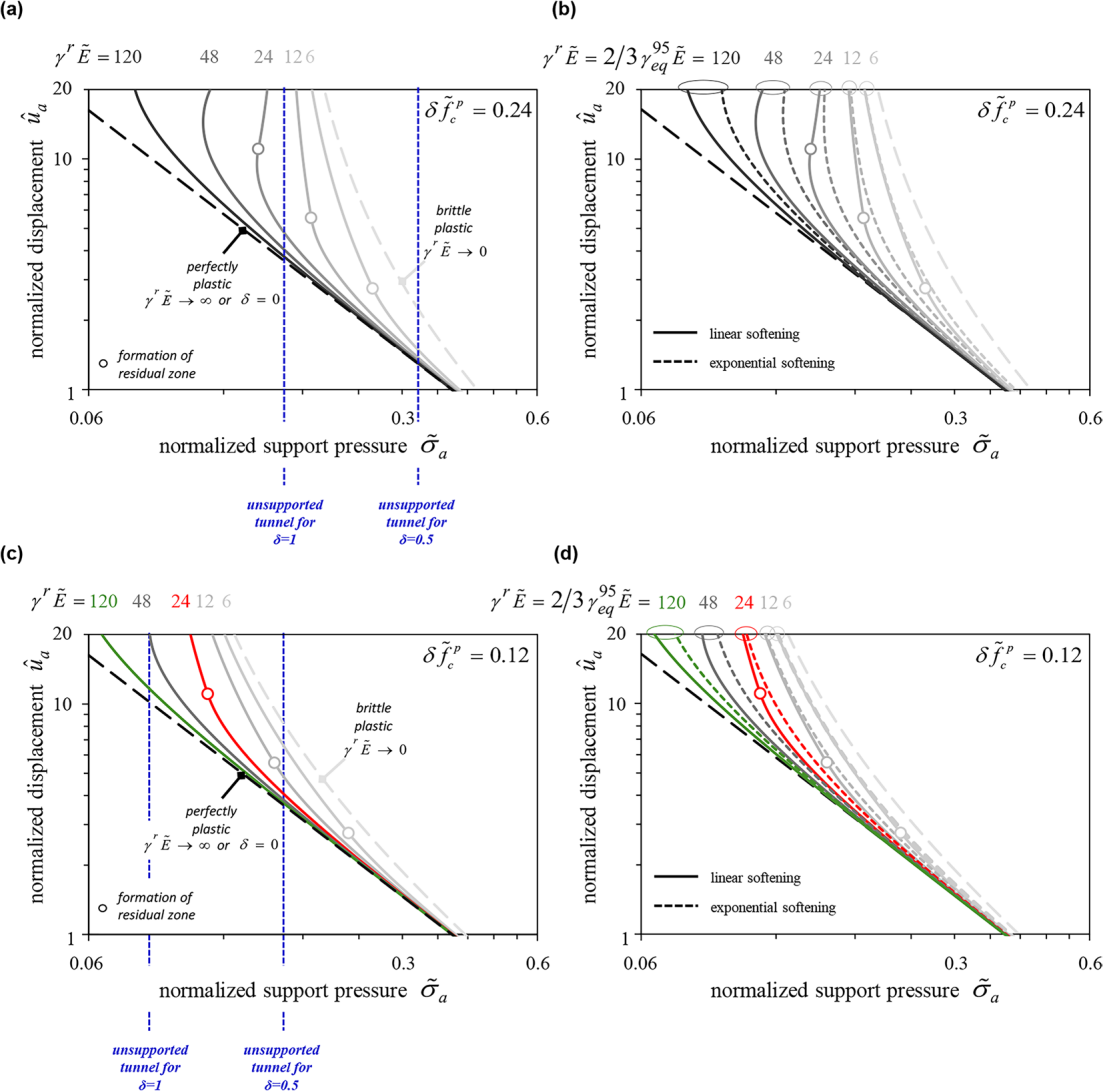
(**a**), (**c**) Normalized GRCs assuming linear strength decay with $\delta \tilde{f}_{c}^{p}$ = 0.24 and 0.12; (**b**), (**d**) Comparison between linear and exponential decay for $\delta \tilde{f}_{c}^{p}$ = 0.24 and 0.12 (MC material, simplifying assumption B, *ζ* = 1; *φ* = 25°, *ψ* = 5°, *ν* = 0.3).

Figure 4a and c illustrate the effect of the two softening parameters $\delta \tilde{f}_{c}^{p}$ and $\gamma^{r} \tilde{E}$, presenting normalized GRCs for a bigger (upper diagram) and a smaller (lower diagram) strength decay ($\delta \tilde{f}_{c}^{p}$ = 0.24 and 0.12 respectively) and a series of $\gamma^{r} \tilde{E}$– values. The dimensionless parameter $\gamma^{r} \tilde{E}$ expresses how rapidly strength decreases with plastic deformations. The GRCs have been obtained considering MC yield criterion with linear strength decay, a cylindrical cavity (*ζ* = 1), *φ* = 25º, *ψ* = 5º and *ν* = 0.3, and making the simplifying Assumption B; comparisons with results for Assumption A and the rigorous solution are given in Section “Error of the simplified solutions”.

The uppermost curve of every diagram applies to the limiting case of a brittle plastic material characterized by a sudden decrease in strength as soon as the strain exceeds the strain at the peak strength. The lowermost line applies to the limiting case of a perfectly plastic material and is identical with Kovári’s GRC^17^ (a straight line in a double logarithmic diagram). With decreasing $\delta \tilde{f}_{c}^{p}$-value (Fig. 4c), the effect of $\gamma^{r} \tilde{E}$ becomes predictably smaller as all GRCs move towards the GRC of the perfectly plastic material (lowest curve).

**Figure 5:**
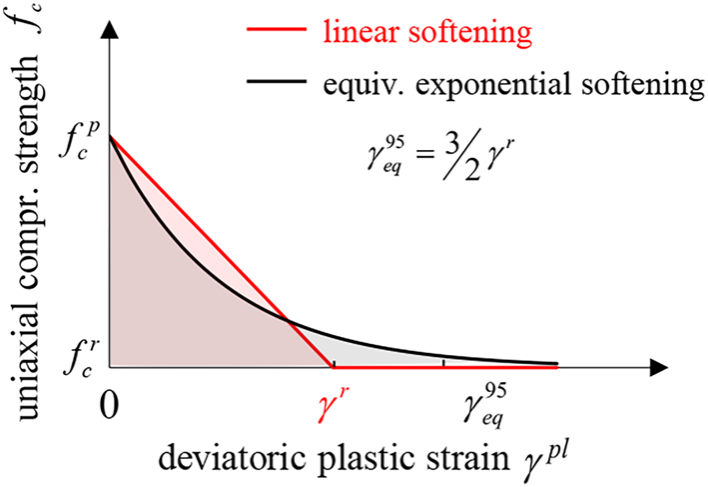
Linear vs exponential softening law for equivalent values of $\gamma^{r}$ and $\gamma^{95}$.

Figure 4b and d show the effects of the softening law on the GRC. The solid lines apply to the linear decay model, the dashed lines to the exponential model. To ensure a meaningful comparison between the two models, an equivalent $\gamma^{95}$ (exponential model) was calculated for every $\gamma^{r}$ (linear model). The estimate of $\gamma_{eq}^{95}$ = 3/2 $\gamma^{r}$ results from the equalization of the integrals of the two functions, *i.e*. of the plastic works expended during shearing (shaded areas in Fig. 5). The exponential model with $\gamma_{eq}^{95}$ shows a faster initial reduction in strength and therefore predicts larger deformations than the linear model. However, the difference is moderate and diminishes with decreasing $\delta \tilde{f}_{c}^{p}$. As expected, the two models converge in the limit cases of perfect plasticity and brittle softening.

Due to the Caquot’s stress-transformation, the GRCs extend to the range of negative support pressures which have no practical significance. Figure 4a and c show by means of vertical blue dashed lines the normalized support pressure $\tilde{\sigma }_{a}$ at which the support pressure $\sigma_{a}$ becomes equal to zero for two cases concerning the post-peak strength decay: a partial strength loss (*δ* = 0.5) and a total strength loss (*δ* = 1). In both Figures, for *δ* = 0.5, all GRCs intersect the blue dashed lines, which means that the convergence of an unsupported opening stabilizes to a finite value, despite the decrease in strength. This is true for *δ* = 1, too, provided that $\gamma^{r} \tilde{E}$ is so big (e.g. 48 and 120 in Fig. 4c) that a residual zone does not form. Otherwise, i.e. if a residual zone with zero uniaxial compressive strength develops, equilibrium is not possible^16^ and, therefore, the displacements increase asymptotically to infinity as the support pressure tends to zero (*e.g*. the lines for $\gamma^{r} \tilde{E}$ ≤ 48 in Fig. 4c).

**Figure 6:**
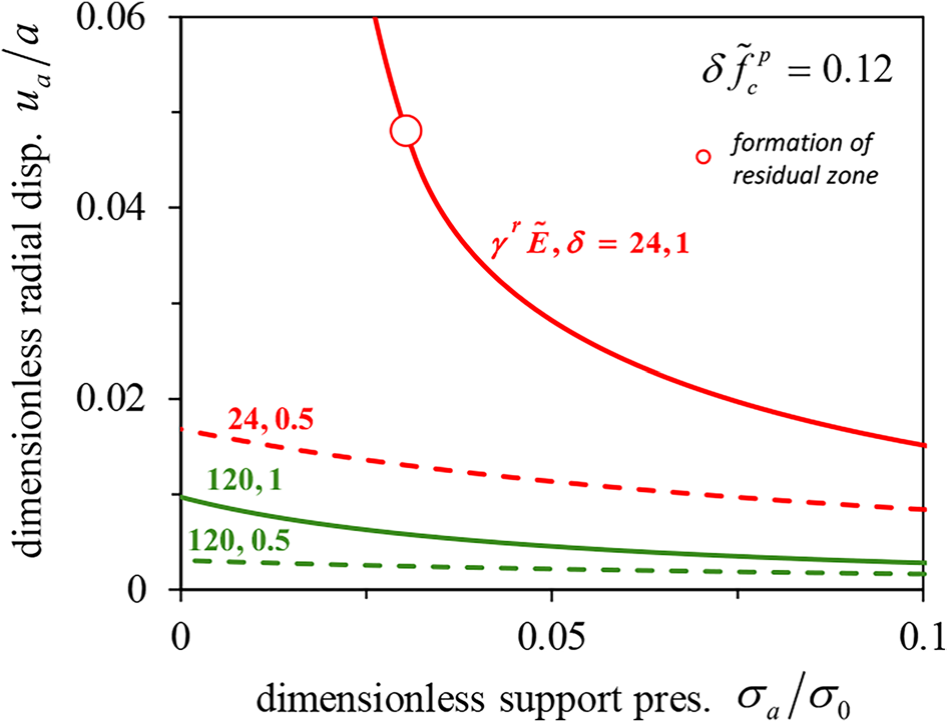
Ground response curves (MC material with linear strength decay, simplifying assumption B, *φ* = 25°, *ψ* = 5°, *ν* = 0.3).

The fact that in case of complete strength loss (*δ* = 1), equilibrium in the unsupported tunnel is not possible if a residual zone is formed, is evident from the GRCs of Fig. 6, where we refrain from Caquot’s transformation in order to visualize better the behaviour at zero support pressure. The curves correspond to the normalized GRCs of the same colour of Fig. 4c, for specific values of $\sigma_{0}$ and *E* or $\gamma^{r}$. The unsupported opening would be unstable in the case of the red solid line (*δ* = 1, $\gamma^{r} \tilde{E}$ = 24) and stable otherwise (*i.e.* in the case of smaller *δ* and/or greater $\gamma^{r} \tilde{E}$). So, one may say that the normalized diagrams of Fig. 4 present in a unified way GRCs for cases that are qualitatively different from the constructional viewpoint (stable vs. unstable unsupported openings).

The stability condition of an unsupported opening in case of potentially total strength loss (*δ* = 1) is *σ*_*b*_ < 0, and can be determined in terms of $\gamma^{r} \tilde{E}$ and peak strength ($\tilde{f}_{c}^{p}$) based upon Eqs. (21) and (23), and it reads as follows:

32 $$\tilde{f}_{c}^{p} \ge \frac{(m - 1)(\zeta + 1)}{{\zeta m + 1}}\left\{ {\begin{array}{*{20}c} 1 & {{\text{if x}} \le {1,}} \\ {\frac{1 + \zeta (m + \kappa - 1)}{{(1 + \zeta \kappa )(x^{\zeta (m - 1)/(\zeta \kappa + 1)} + \zeta (m - 1)x^{ - 1} }}} & {{\text{if x}} > 1{,}} \\ \end{array} } \right.$$

where

33 $$x = \frac{{\gamma^{r} \tilde{E}}}{{(1 + \zeta \kappa )\hat{\varepsilon }_{t,\rho }^{el} }}.$$

**Figure 7:**
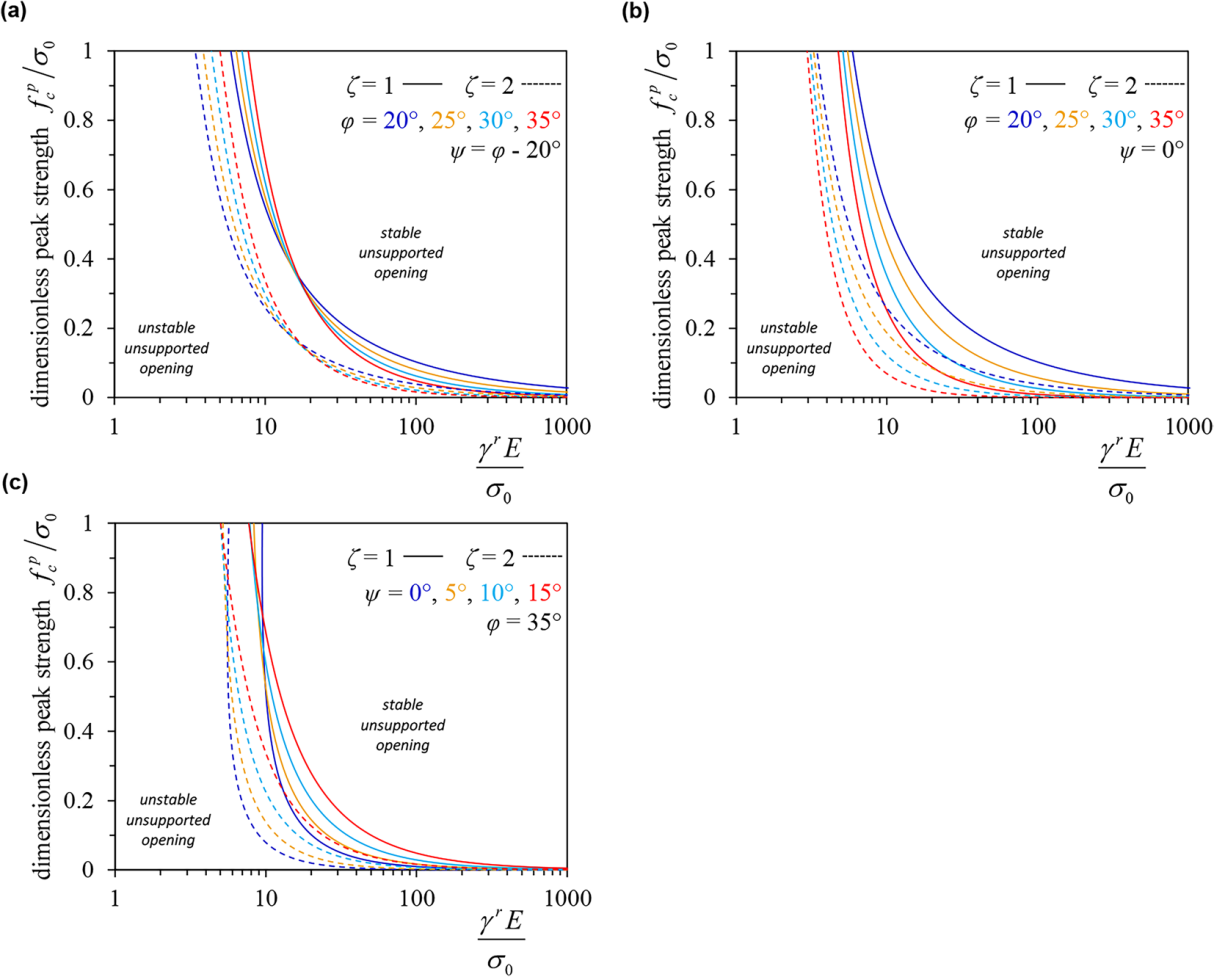
Stability condition for, (**a**), varying *φ* and *ψ*, (**b**), varying *φ* and, (**c**), varying *ψ* (MC material with linear strength decay, simplifying assumption B, *ν* = 0.3).

Figure 7a shows this condition for varying values of *φ* (20°, 25°, 30°, and 35°) and *ψ*, where *ψ* is taken equal to *φ*—20°. It is interesting that an elasticity parameter, the Young’s modulus, is decisive for the stability (or lack thereof) of an unsupported opening. This is because the Young’s modulus affects the plastic strains (the latter increase linearly with 1/*E* in the case of a perfectly plastic material^30^) and thus, in the case of softening, how much strength is lost. The opening type (cylindrical tunnel or spherical cavity) has a small effect on the stability condition. In Fig. 7b and c, the friction and dilation angles, respectively, are independently varied. An increased friction angle is advantageous for the stability of the unsupported opening, while on the contrary, an increased dilation results in larger plastic strains, leading to a more significant loss of strength in the case of a softening material. However, both *φ* and *ψ* have a relatively minor impact on stability, which justifies the simplification of keeping them constant in the strain softening regime.

## Error of the simplified solutions

To assess the error of the simplified solutions, we employ the numerical model proposed by Lee and Pietruszczak^26^. This model enables the calculation of stress, strain, and displacement fields around a circular tunnel excavated in a strain-softening material. The plastic zone is discretized into a finite number of concentric rings and their thickness is determined to ensure equilibrium and compatibility. By using a sufficient number of annuli, this method accurately approximates the rigorous closed-form solutions.

**Figure 8:**
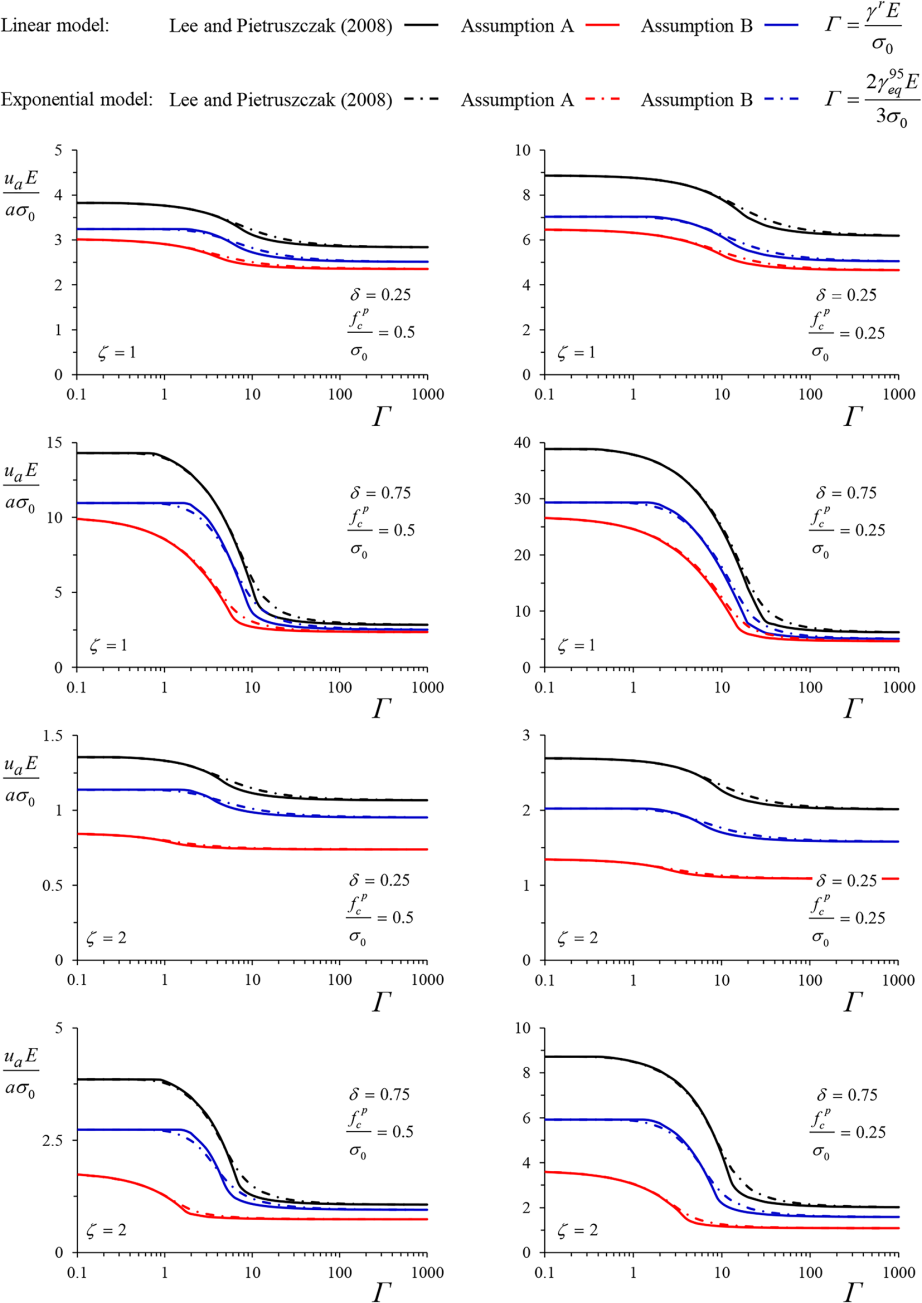
Normalized convergence of an unsupported cavity over the dimensionless factor ${{\gamma^{r} E} \mathord{\left/ {\vphantom {{\gamma^{r} E} {\sigma_{0} }}} \right. \kern-0pt} {\sigma_{0} }}$ for the linear model and ${{2\gamma_{eq}^{95} E} \mathord{\left/ {\vphantom {{2\gamma_{eq}^{95} E} {\left( {3\sigma_{0} } \right)}}} \right. \kern-0pt} {\left( {3\sigma_{0} } \right)}}$ for the exponential model, after^26^ and according to the two simplified solutions (MC material, *φ* = 25°, *ψ* = 5°, *ν* = 0.3).

**Figure 9:**
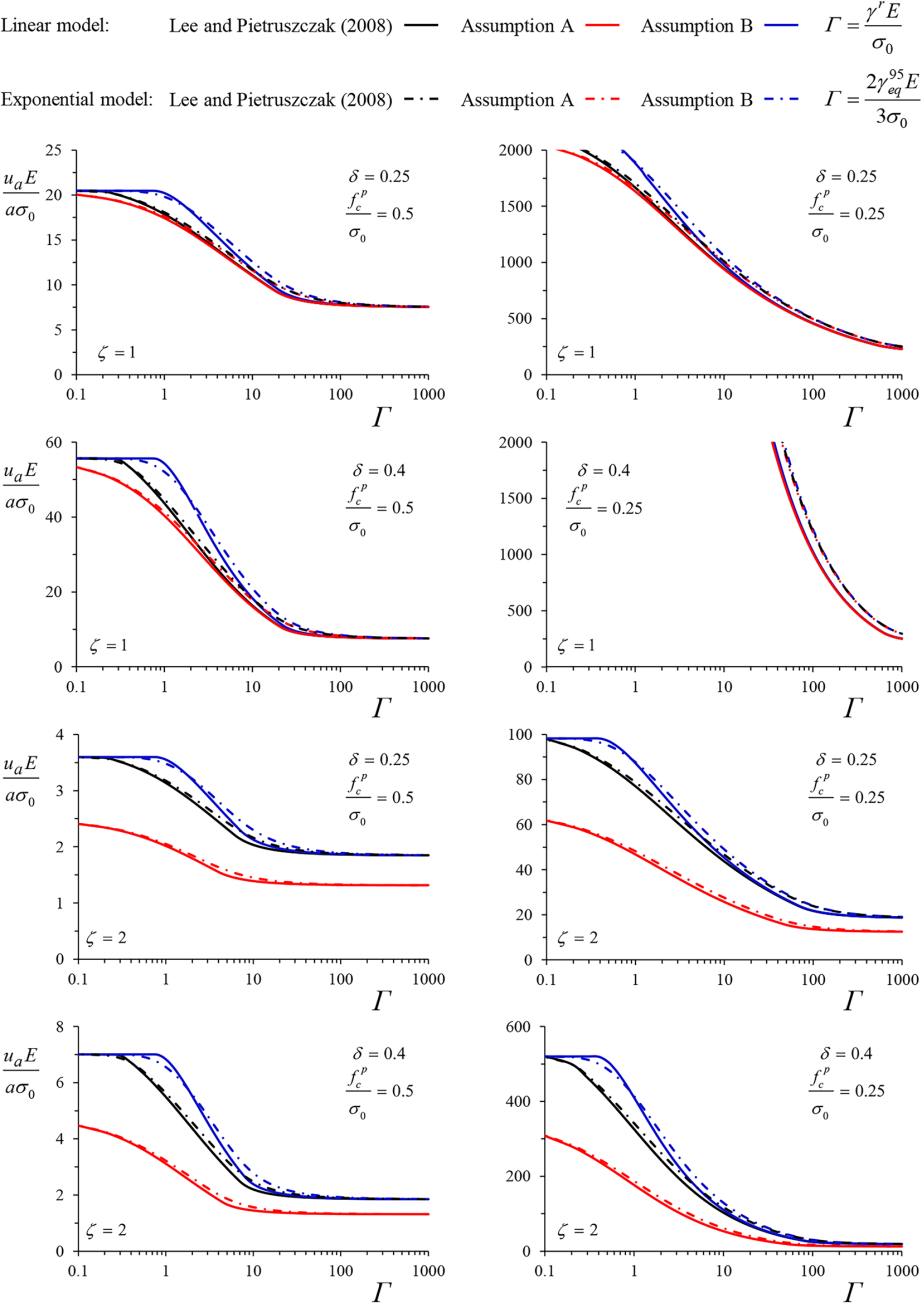
Normalized convergence of an unsupported cavity over the dimensionless factor ${{\gamma^{r} E} \mathord{\left/ {\vphantom {{\gamma^{r} E} {\sigma_{0} }}} \right. \kern-0pt} {\sigma_{0} }}$ for the linear model and ${{2\gamma_{eq}^{95} E} \mathord{\left/ {\vphantom {{2\gamma_{eq}^{95} E} {\left( {3\sigma_{0} } \right)}}} \right. \kern-0pt} {\left( {3\sigma_{0} } \right)}}$ for the exponential model, after^26^ and according to the two simplified solutions (Tresca material).

Figures 8 and 9 present the normalized convergence of an unsupported cavity for the cases of the MC and Tresca yield criteria, respectively. In both figures, the upper four diagrams hold for cylindrical cavities (*ζ* = 1) and the lower four diagrams for spherical cavities (*ζ* = 2). We investigate two values of the normalized peak strength, characterizing two squeezing intensities: moderate squeezing (*f*_*c*_^*p*^*/σ*_*0*_ = 0.5, left diagrams) and heavy squeezing (*f*_*c*_^*p*^*/σ*_*0*_ = 0.25, right diagrams). Two values for the maximum strength loss are considered: a smaller one, *δ* = 0.25, and a bigger one, *δ* = 0.75 for the MC material and 0.4 for the Tresca material. In the latter, a more unfavourable value of *δ* was not examined, since it would result in large strains and displacements that lack physical meaning and undermine the validity of the small-strain assumption, underlying both the analytical and the numerical methods.

Each diagram in Figs. 8 and 9 presents the dimensionless convergence as a function of *Γ, i.e.,* ${{\gamma^{r} E} \mathord{\left/ {\vphantom {{\gamma^{r} E} {\sigma_{0} }}} \right. \kern-0pt} {\sigma_{0} }}$ for the linear model and ${{2\gamma_{eq}^{95} E} \mathord{\left/ {\vphantom {{2\gamma_{eq}^{95} E} {\left( {3\sigma_{0} } \right)}}} \right. \kern-0pt} {\left( {3\sigma_{0} } \right)}}$ for the exponential model. For each model, the following three curves are given; the rigorous solution based on the numerical method by Lee and Pietruszczak^26^ (black curves) and the simplified solutions presented in this paper based on Assumption B (blue curves), and Assumption A (red curves), as derived from Eqs. (21), (22), (26) and (27).

For the MC material (Fig. 8), both simplified solutions underestimate the convergence of the unsupported tunnel, with the proposed solution (blue curves) consistently improving Assumption A—based solutions (red curves). The improvement is particularly significant for the spherical cavity. Each curve approaches the solution for brittle plastic materials asymptotically for small values of *Γ* and the solution for perfectly plastic materials for big values of *Γ*. The difference between the linear and the exponential model is insignificant and, as expected, visible only for intermediate values of *Γ*. Apparently, the numerical results (black curves) tend towards the exact borderline solutions, while the analytical results (red and blue curves) tend towards the simplified ones.

For the Tresca material (Fig. 9) and a cylindrical cavity (upper four diagrams), both simplified solutions closely approximate the exact one, with Assumption A slightly underestimating the convergence and Assumption B slightly overestimating it. Both simplified solutions converge towards the numerical one, as the analysis approaches the borderline perfectly-plastic and brittle-plastic cases, which are, in this case, unaffected by the simplifying assumption. Nevertheless, the difference is significant for the spherical cavity. While Assumption B only slightly overestimates the convergence, Assumption A greatly underestimates it. The latter is due to the violation of the zero volumetric condition, as discussed in the Introduction.

**Figure 10:**
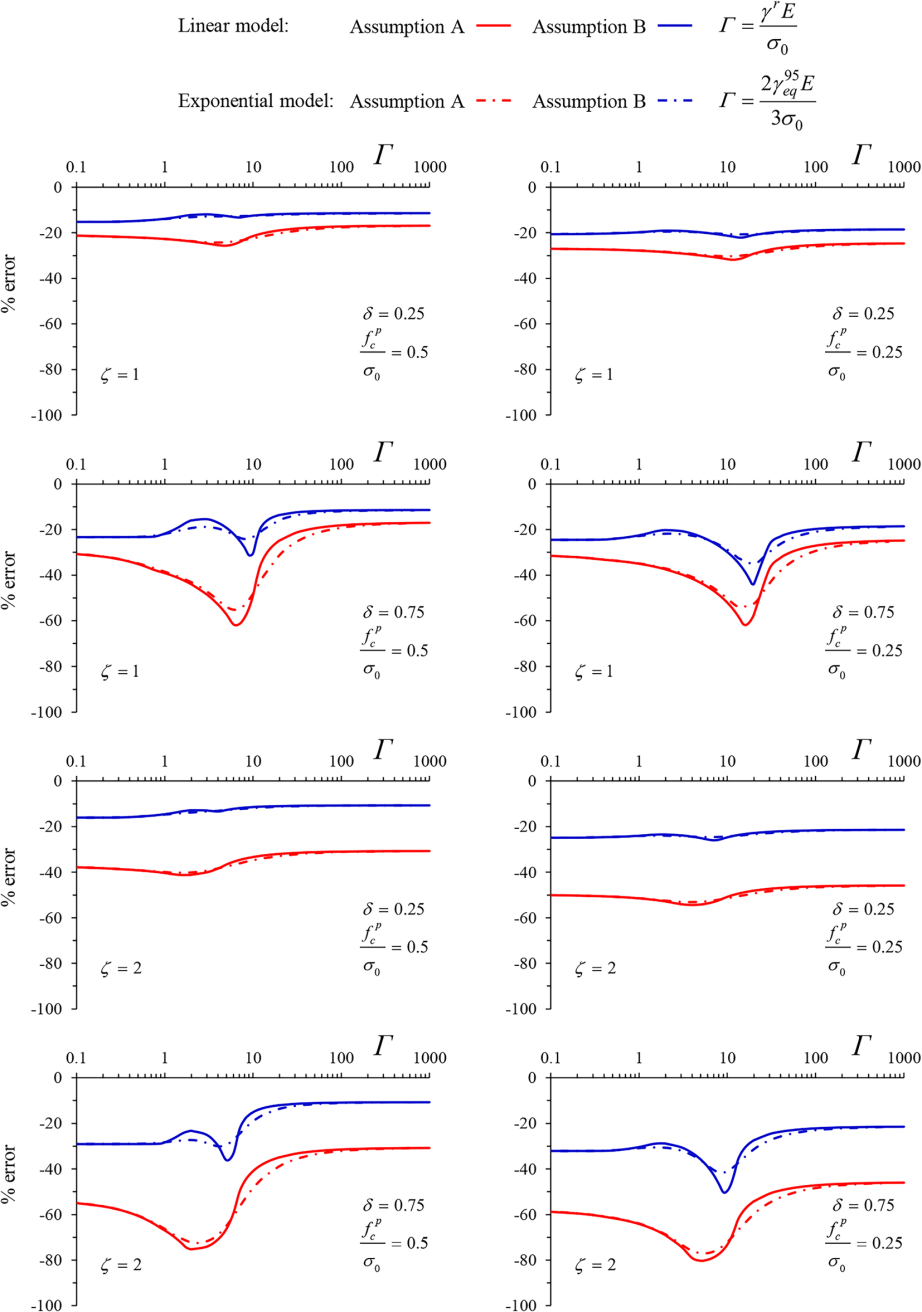
Percentage error in radial displacement for the two simplified solutions (MC material).

**Figure 11:**
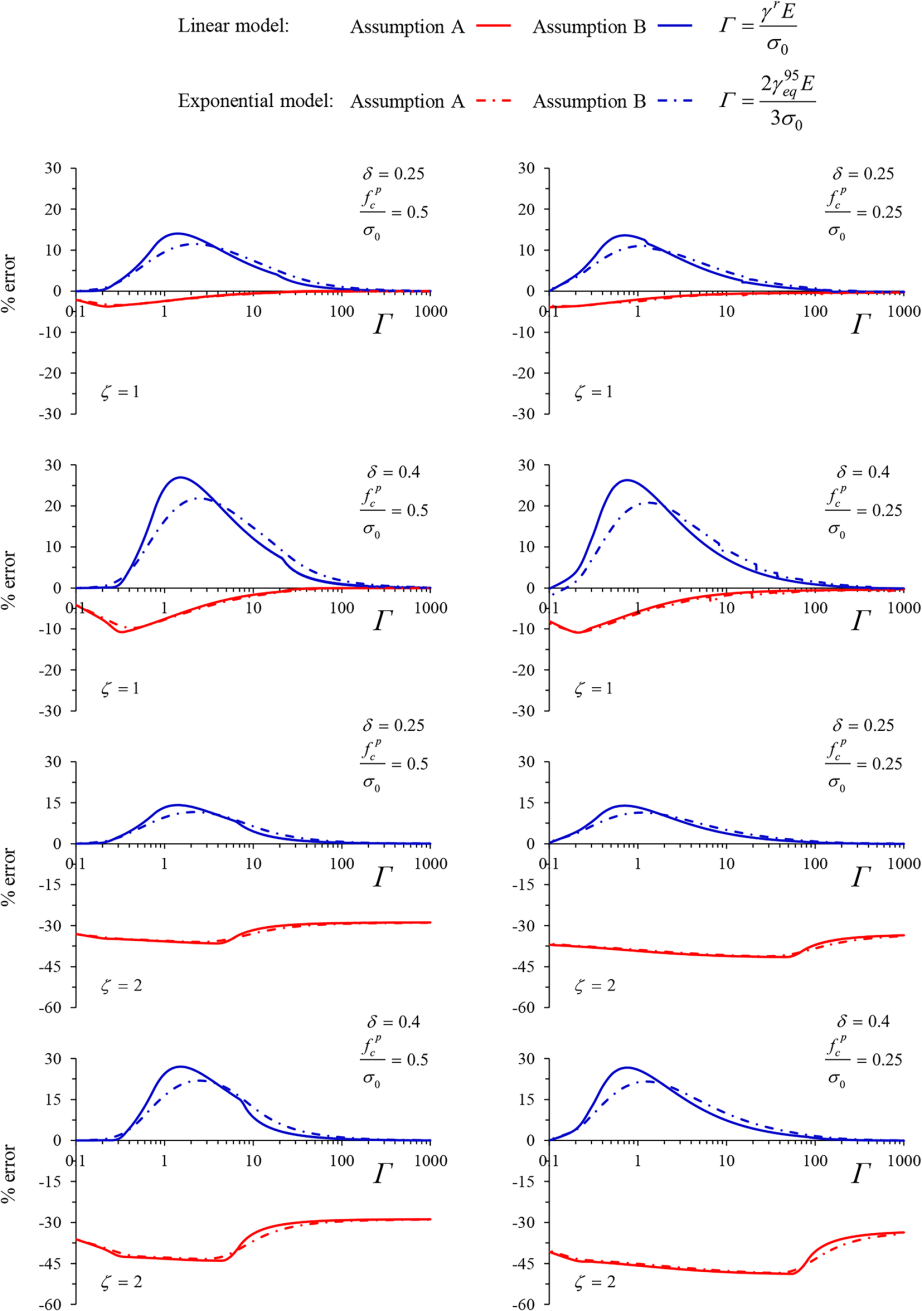
Percentage error in radial displacement for the two simplified solutions (Tresca material).

Figures 10 and 11 present the percentage error in radial displacement for the two simplified solutions, for MC and Tresca materials, respectively.

Despite the non-negligible errors present in both solutions, the proposed one (blue curves) consistently demonstrates reduced errors, remaining below 30% in most cases. An exemption occurs in the case of the cylindrical tunnel for the Tresca material, where the error of the proposed solution exceeds that of the Assumption A—based one.

However, unlike the Assumption A—based solution, the proposed one lies on the safe side. The error peaks for intermediate values of *Γ*, corresponding to the steep part of the curves depicted in Figs. 8 and 9. The maximum error is, in all cases, slightly smaller for the exponential model.

## Conclusions

In this study, analytical closed-form expressions were derived for assessing the convergence of cylindrical and spherical cavities excavated in strain-softening rock masses. The analysis considered two yield criteria, the Mohr–Coulomb and the Tresca one, with linear and exponential strength decay within the strain-softening range. It was assumed that the decrease in strength is governed by the plastic deviatoric strain, as suggested in the literature.

The comparison of the linear and exponential models for equivalent values of $\gamma^{r}$ and $\gamma^{95}$, revealed that the exponential model is less favourable, but the difference is moderate. For materials following the MC criterion, the GRCs were expressed in a normalized form using Caquot’s stress transformation. This enables the inclusion of qualitatively different cases, *i.e*. stable and unstable unsupported openings, in the same GRC. Specifically, for given cavity support pressure and strength drop *δ*, stable conditions are characterized by a finite dimensionless convergence of the cavity. Otherwise, the convergence tends towards infinity when the support pressure approaches zero. In the case of complete loss of strength, an unsupported tunnel cannot be stable. The stability condition for the latter case was presented in terms of peak uniaxial strength and Young’s modulus normalized to *σ*_*0*_, and deviatoric plastic strain to reach the residual state.

The derivation of closed-form GRCs was made possible by adopting a simplifying assumption for the strains inside the plastic zone. Two alternative assumptions were examined: Assumption A, commonly employed in the literature for strain-softening materials^16,24^, neglects the elastic strains developed during plastic yielding; Assumption B, previously used for perfectly plastic and brittle plastic materials (^17,19^), completely disregards the elastic strains within the plastic zone. For materials following the MC criterion, it was shown that the solution based on Assumption B consistently outperforms the one based on Assumption A, while both underestimate the tunnel convergence compared to rigorous results obtained from the finite—difference model suggested by Lee and Pietruszczak^26^. For the Tresca material and cylindrical cavities, both analytical solutions converge to the numerical one, with the proposed solution exhibiting slightly bigger errors, but on the safe side. A notable difference is observed for spherical cavities, where the solution based on Assumption B slightly overestimates the response, while Assumption A significantly underestimates it. The linear and the exponential model, for equivalent values of $\gamma^{r}$ and $\gamma^{95}$, predict similar convergences, but the exponential model exhibits a slightly smaller maximum error than the linear.

## Data Availability

The datasets used and analysed during the current study are available from the corresponding author on reasonable request.

## Latin symbols

*a*: Tunnel radius
*b*: Radius of residual strength zone
*E*: Young’s modulus
$\tilde{E}$: Normalized Young’s modulus
*f*_*c*_: Uniaxial compressive strength
$f_{c}^{p}$: Peak uniaxial compressive strength
$\tilde{f}_{c}^{p}$: Normalized peak uniaxial compressive strength
*f*_*c*_^*r*^: Residual uniaxial compressive strength
*m*: Friction strength factor
*n*: Exponential strength decay factor
*r*: Radial coordinate
*u*: Radial displacement
*u*_*a*_: Radial displacement of the cavity wall
$\hat{u}_{a}$: Normalized radial displacement of the cavity wall

## Greek symbols

*γ*^*pl*^: Deviatoric plastic strain
*γ*^*r*^: Deviatoric plastic strain to reach residual state
*γ*^*95*^: Deviatoric plastic strain to reach 95% strength decay
*γ*_*eq*_^*95*^: Equivalent deviatoric plastic strain of the exponential model
*Γ*: Dimensionless deviatoric plastic strain factor
*δ*: Maximum decrease in strength normalized by the peak strength
*ε*_*r*_: Radial strain
$\varepsilon_{r}^{pl}$: Radial plastic strain
$\varepsilon_{r,\rho }^{el}$: Radial elastic strain at the elasto-plastic boundary
*ε*_*t*_: Tangential strain
$\varepsilon_{t}^{pl}$: Tangential plastic strain
$\varepsilon_{t,\rho }^{el}$: Tangential elastic strain at the elasto-plastic boundary
$\hat{\varepsilon }_{t,\rho }^{el}$: Normalized tangential elastic strain at the elasto-plastic boundary
*ζ*: Cavity symmetry parameter
*κ*: Loosening factor
*λ*: Exponential strength decay factor
*μ*: Linear strength decay factor
*ν*: Poisson’s ratio
*ρ*: Radius of plastic zone
$\tilde{\rho }$: Normalized radius of plastic zone
$$\tilde{\sigma }$$: Normalized stress
*σ*_*a*_: Tunnel support pressure
$\tilde{\sigma }_{a}$: Normalized tunnel support pressure
*σ*_*b*_: Stress at the softening-residual boundary
$\tilde{\sigma }_{b}^{{}}$: Normalized stress at the softening-residual boundary
*σ*_*r*_: Radial stress
*σ*_*t*_: Tangential stress
*σ*_*ρ*_: Radial stress at the elastic boundary
*σ*_*0*_: In-situ stress
$\overline{\sigma }_{0}$: Transformed in-situ stress
*φ*: Friction angle
*ψ*: Dilation angle
*ω*: Normalized radius of plastic zone for which residual zone is formed
